# Host Specificity of the *Dickeya* Bacteriophage PP35 Is Directed by a Tail Spike Interaction With Bacterial *O*-Antigen, Enabling the Infection of Alternative Non-pathogenic Bacterial Host

**DOI:** 10.3389/fmicb.2018.03288

**Published:** 2019-01-11

**Authors:** Anastasia P. Kabanova, Mikhail M. Shneider, Aleksei A. Korzhenkov, Eugenia N. Bugaeva, Kirill K. Miroshnikov, Evelina L. Zdorovenko, Eugene E. Kulikov, Stepan V. Toschakov, Alexander N. Ignatov, Yuriy A. Knirel, Konstantin A. Miroshnikov

**Affiliations:** ^1^Shemyakin-Ovchinnikov Institute of Bioorganic Chemistry, Russian Academy of Sciences, Moscow, Russia; ^2^Research Center “PhytoEngineering” Ltd., Rogachevo, Russia; ^3^Immanuel Kant Baltic Federal University, Kaliningrad, Russia; ^4^Winogradsky Institute of Microbiology, Federal Research Center “Fundamentals of Biotechnology”, Russian Academy of Sciences, Moscow, Russia; ^5^Zelinsky Institute of Organic Chemistry, Russian Academy of Sciences, Moscow, Russia

**Keywords:** bacteriophage, *Dickeya solani*, *Lelliottia*, genomics, tail spike protein, polysaccharide, depolymerase

## Abstract

*Dickeya solani* is a recently emerged virulent bacterial potato pathogen that poses a major threat to world agriculture. Because of increasing antibiotic resistance and growing limitations in antibiotic use, alternative antibacterials such as bacteriophages are being developed. *Myoviridae* bacteriophages recently re-ranked as a separate *Ackermannviridae* family, such as phage PP35 described in this work, are the attractive candidates for this bacterial biocontrol. PP35 has a very specific host range due to the presence of tail spike protein PP35 gp156, which can depolymerize the *O*-polysaccharide (OPS) of *D. solani*. The *D. solani* OPS structure, →2)-β-D-6-deoxy-D-altrose-(1→, is so far unique among soft-rot *Pectobacteriaceae*, though it may exist in non-virulent environmental *Enterobacteriaceae*. The phage tail spike depolymerase degrades the shielding polysaccharide, and launches the cell infection process. We hypothesize that non-pathogenic commensal bacteria may maintain the population of the phage in soil environment.

## Introduction

Soft-rot *Pectobacteriaceae* (SRP) include phytopathogenic bacterial species from the genera *Pectobacterium* and *Dickeya* that cause economic losses in potato crops, as well as other vegetables and ornamental plants worldwide (Pérombelon, 2002). *Dickeya* spp. were mostly associated with plant diseases in tropical climates (Toth et al., 2011). A new virulent species named *D. solani* emerged in the early 2010s (Laurila et al., 2010; van der Wolf et al., 2014) and rapidly spread throughout Europe, including Russia (Ignatov et al., 2014). No potato cultivars is resistant to *D. solani*, and the spread of the disease is mostly contained by quarantining and controlling seed stocks (Mansfield et al., 2012). Treating seeds and harvested tubers with bacteriophages – viruses specific to particular bacterial pathogens – is considered a promising and environmentally safe strategy to protect plants and harvested crops from bacterial diseases (Frampton et al., 2012). A number of isolated bacteriophages infect *D. solani* (Adriaenssens et al., 2012b; Day et al., 2017) and some can also infect other *Dickeya* spp. (Czajkowski et al., 2014b, 2015). They have been thoroughly characterized and used to protect and control soft rot caused by *D. solani.* The primary goal of the present study was an investigation of the *D. solani –* specific bacteriophage newly isolated in Russia and the molecular details of its interaction with the bacterial host.

## Materials and Methods

### Isolation and Purification of Phage PP35

Bacteriophage PP35 was isolated in 2014 from sewage water near an outbreak of soft rot in harvested potatoes caused by *D. solani* (Moscow region, Russia). Phage was propagated on *D. solani* strain F012 in LB at 26°C following a published general protocol (Clokie and Kropinski, 2009). After chloroform treatment, removal of cell debris by centrifugation (8000 *g*, 20 min), filtration through a 0.22-μm-pore size membrane filter (Millipore), and treatment with DNase I (0.5 mg/mL, 60 min), the phage was purified by ultracentrifugation (22,000 *g*, 120 min, 4°C, Beckman SW28 rotor) in a CsCl step gradient with densities of 0.5 to 1.7 g/mL. The resulting suspension of PP35 was dialyzed overnight against phage buffer (10 mM Tris–HCl, pH 7.4, 10 mM MgSO_4_) to remove CsCl. Purified phage was stored at 4°C in phage buffer.

### Host Range and General Characterization of Phage PP35

The host range of phage PP35 was tested by standard plaque assays and by spotting phage suspensions (10^6^ pfu/ml) onto a bacterial lawn. Bacterial strains listed in Supplementary Table S1 were grown on LB agar at 26°C. In adsorption experiments, the host strains F012 or F154 were grown to an OD_600_ ∼ 0.4 and infected with PP35 at a multiplicity of infection of 0.1. Every 3 min after infection, 100 μl aliquots were taken and transferred into 800 μl LB medium supplied with 50 μl chloroform. After bacterial lysis the mixtures were centrifuged and the supernatant was titrated to determine the amount of non-adsorbed or reversibly adsorbed phages. One-step-growth assays were performed according to Adriaenssens et al. (2012b). To assay a lytic activity of phage PP35, an exponentially growing culture of F012 or F154 (10^6^ cfu/ml) was mixed with phage PP35 (MOI of 0.1). The mixture was then incubated with shaking at 26°C. Every 10 min, aliquots were taken, and the appropriate dilutions were spread on LB agar plates, and incubated overnight at 26°C. The next day, colonies were counted. Phage stability was studied by incubating a 10^7^ pfu/ml phage suspension at different temperatures or in a range of buffer solutions (20 mM Tris–HCl/20 mM Na citrate/20 mM Na phosphate) adjusted with NaOH to pH range 4–9. All experiments were performed independently 3–4 times, and the results were averaged. Plots were generated with Microsoft Excel.

### Electron Microscopy

Purified phage particles were applied to grids and stained with 1% uranyl acetate aqueous solution (Ackermann, 2009). The specimens were observed in a JEOL JEM-CX100 electron microscope at 100 kV accelerating voltage.

### Genome Sequencing and Annotation

Bacterial and phage DNA was extracted using the phenol-chloroform method and fragmented with a Bioruptor sonicator (Diagenode). Paired-end libraries were constructed using Nebnext Ultra DNA library prep kit (New England Biolabs) and sequenced on the Illumina MiSeq^TM^ platform (Illumina) using paired 150 bp reads. After filtering with CLC Genomics Workbench 8.5 (Qiagen), overlapping paired-end library reads were merged with the SeqPrep tool^1^. Reads from bacteriophage PP35 were assembled in CLC Genomic workbench v. 7.5; reads from F012 and F154 bacterial strains were assembled using SPAdes 3.6.1 (Bankevich et al., 2012). Contig count, assembly length and mean coverage are displayed in Table 1.

**Table 1:** Genome assembly properties.

Genome	Contig (scaffold) count	Assembly length, bp	Mean coverage
PP35 (phage)	1 (1)	152 048	245
*Dickeya* F012	27 (25)	4 879 104	52.3
*Lelliottia* F154	23 (23)	4 457 928	54.3


Bacterial genomes were annotated with the RAST automated pipeline^2^ (Aziz et al., 2008). Phage genome was annotated by first calling ORFs with GeneMarkS (Besemer et al., 2001). ORF functions were predicted using Psi-BLAST alignment against the NCBI nr database (Altschul et al., 1997) and Pfam domain prediction using HMMER (Finn et al., 2011). tRNA coding regions were identified with tRNAscan-SE (Schattner et al., 2005), search of other non-coding RNA was performed using SEED. Putative phage promoters were predicted by PHIRE (Lavigne et al., 2004) and phiSITE (Klucar et al., 2009). CRISPR arrays were detected using CRISPRfinder^3^. ANI were calculated using the ani.rb script^4^. Genome sequences were clustered by ANI value within R.

### Phage Genome Comparison and Taxonomy

A phylogeny for the phage was constructed using the VICTOR server (Meier-Kolthoff and Göker, 2017). All pairwise comparisons of the amino acid sequences were conducted using the Genome-BLAST Distance Phylogeny (GBDP) method (Meier-Kolthoff et al., 2013) with settings recommended for prokaryotic viruses (Meier-Kolthoff and Göker, 2017). The resulting intergenomic distances were used to infer a balanced minimum evolution tree with branch support via FASTME including SPR post-processing for each of the formulas D0, D4, and D6, respectively (Lefort et al., 2015). Branch support was inferred from 100 pseudo-bootstrap replicates each. Trees were rooted at the midpoint (Farris, 1972) and visualized with FigTree (Rambaut, 2016). Taxon boundaries at the species, genus, and family level were estimated with the OPTSIL program, the recommended clustering thresholds (Meier-Kolthoff and Göker, 2017) and an *F* value (fraction of links required for cluster fusion) of 0.5.

Sequences were uploaded into the ANI calculator package^5^ in order to perform pairwise genome calculations of the ANI using the default conditions. The ANI calculator estimates the average nucleotide identity using reciprocal best hits (two-way ANI) between two genomic datasets.

### Tail Spike Protein Cloning, Expression and Purification

Nucleotide sequence representing phage PP35 ORF156 (120600–122246) was PCR-amplified using primers 5′-TATTTCCAGGGCAGCGGATCCAACTCGCAATTCTCACAGCCG (forward) and 5′-GCTCGAGTGCGGCCGCAAGCTTACCCCAATTTGTACCAGG (reverse) bearing BamHI and HindIII restriction sites. An amplicon was cloned to vector pTSL (Taylor et al., 2016) using NEBuilder HiFi DNA Assembly kit (New England Biolabs). Clones with inserts were selected by PCR, restriction analysis, and verified by sequencing. Preparative gene expression was performed in *E. coli* B834(DE3) by induction with 1 mM IPTG at 16°C overnight. Cells were pelleted at 4,000 g, lysed by sonication in lysis buffer (20 mM Tris–HCl (pH 8.0), 200 mM NaCl), and centrifuged (13,000 *g*) to remove debris. Recombinant tail spike protein (PP35gp156) was purified on a Ni-NTA Sepharose column (GE Healthcare, 5 mL) by a stepwise gradient from 0 to 200 mM imidazole in 20 mM Tris–HCl (pH 8.0), 200 mM NaCl. Purified fractions were dialyzed against 20 mM Tris–HCl (pH 8.0) to remove imidazole, and treated with TEV protease for 12 h at 20°C. The target protein was purified on a 5 mL SourceQ 15 (GE Healthcare) column by a linear gradient of 0–600 mM NaCl in 20 mM Tris–HCl (pH 8.0). Protein concentration was determined spectrophotometrically at 280 nm using a theoretical absorption coefficient of 65,320 M^-1^ cm^-1^. PP35 gp156 oligomeric state was assessed by gel filtration on Superdex 200 10 × 300 column (GE Healthcare).

### Isolation of the *O*-Polysaccharides

Bacterial LPS was isolated by extraction of bacterial cells (∼2 g) with hot phenol-water (Westphal and Jann, 1965) followed by removal of nucleic acids and proteins by precipitation with 50% trichloroacetic acid at 4°C. A LPS sample (70 mg) was hydrolyzed with aqueous 2% HOAc at 100°C for 1 h, a lipid precipitate was removed by centrifugation (13,000 *g*, 20 min), and the carbohydrate portion was fractionated by gel-permeation chromatography on a Sephadex G-50 Superfine (GE Healthcare) column (56 × 2.6 cm) in 0.05 M pyridinium acetate buffer (pH 4.5) monitored with a Knauer differential refractometer to yield a high-molecular-mass OPS preparation (7 mg).

### Treatment of the *O*-Polysaccharides With Bacteriophage PP35 Recombinant Tail Spike Protein

Dried sample of F012 or F154 *O*-polysaccharide was solubilized in 20 mM Tris–HCl (pH 8.0), then purified PP35 gp156 recombinant protein was added to 1/100 (*w*/*w*), and the reaction mixture was incubated overnight at 20°C.

### NMR Spectroscopy of *O*-Polysaccharides

Samples were deuterium-exchanged by freeze-drying twice from 99.9% D_2_O and then examined as solutions in 99.9% D_2_O. ^1^H and ^13^C NMR spectra were recorded on a Bruker Avance II 600 MHz spectrometer (Germany) at 50°C using sodium 3-trimethylsilylpropanoate-2,2,3,3-d_4_ (δ_H_ 0.0, δ_C_ –1.6) as internal reference for calibration. Assignments of the ^1^H and ^13^C NMR signals were measured using two-dimensional ^1^H,^1^H COSY, ^1^H,^1^H TOCSY, and ^1^H,^13^C HSQC experiments, which were done by standard Bruker software. The Bruker TopSpin 2.1 program was used to acquire and process the NMR data. Spin-lock time of 60 ms was used in the ^1^H,^1^H TOCSY experiment.

### Mass Spectrometry of Products of OPS Degradation With Recombinant PP35 gp156

Negative ion mode HR ESI MS was performed on a Bruker micrOTOF II instrument. Interface capillary voltage was 3200 V, mass range from *m/z* 50 to 3000 Da. Samples were dissolved in a 1:1:0.1 acetonitrile/water/triethylamine mixture, and the solution was injected with a syringe at a flow rate of 3 μL/min. Nitrogen was applied as drying gas; interface temperature was set at 180°C. Internal calibration was done with Electrospray Calibrant Solution (Fluka).

## Results

### Basic Properties of Phage PP35

Bacteriophage PP35 was isolated in 2014 from sewage water in a potato storage warehouse (Moscow region, Russia) with a soft rot infection caused by *D. solani*. Pathogenic strain F156 was identified as *D. solani* by PCR amplification and DNA sequencing of the 16S rRNA region (van Vaerenbergh et al., 2012). The better characterized *D. solani* lab strain, F012, was used as a host for phage propagation. Phage PP35 forms small clear plaques 1–2 mm in diameter on strains of *D. solani* including strain F012. We obtained a high phage titer for further purification, genomic DNA extraction, and electron microscopy. Phage PP35 is stable in solution between a pH of 5–10, and temperatures between 4 and 40°C. Freezing without cryoprotectors or heating above 50°C causes phage particles to rapidly lose infectivity.

**FIGURE 1:**
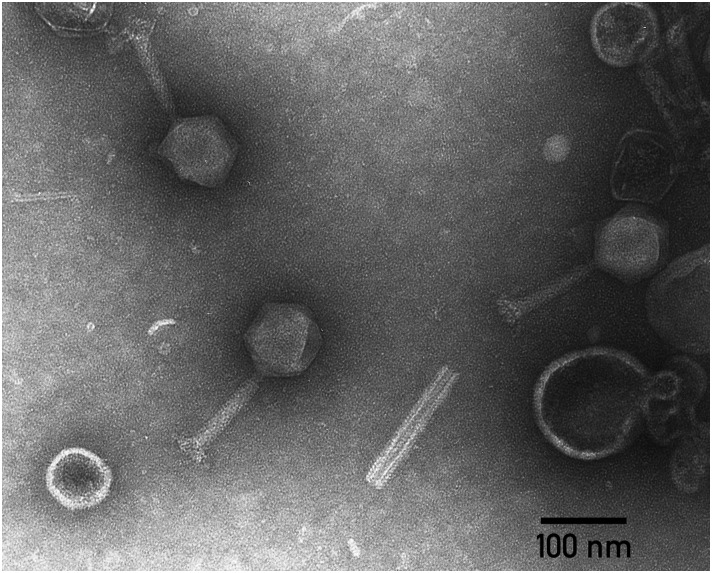
Electron micrograph of PP35 virions. Specimens were stained with 1% uranyl acetate.

Transmission electron microscopy shows that the phage PP35 particle has a contractile tail (∼ 130 nm long), isometric icosahedral head (∼95 nm in diameter), and a pronounced baseplate complex with approximately 10 nm-long tail spikes (Figure 1). The morphology of PP35 is similar to that of *Salmonella* phage Vi1 (Pickard et al., 2010) and other phages assigned to the Vi1-like group. This large group of phages was previously referred as the *Vi1virus* genus of the family *Myoviridae* (Adriaenssens et al., 2012a). However, Vi1-like viruses were reclassified recently as the taxonomic family *Ackermannviridae*, where *Dickeya* viruses related to phage Limestone are grouped as the *Limestonevirus* genus of subfamily *Aglimvirinae* (Adriaenssens et al., 2017). Therefore, dependent on the ratification of the proposal, the unified naming of PP35 should be either vB_DsoM_PP35 or vB_DsoA_PP35.

### Phage Host Range Determination

Among 36 strains representing various *Pectobacterium* and *Dickeya* spp. causing black leg and soft rot in potato, phage PP35 only infects nine strains determined as *D. solani*, and not *D. dianthicola, P. atrosepticum, P. parmentieri, P. carotovorum* subsp. *carotovorum* and *P.c.* subsp. *brasilense* (Supplementary Table S1). This corresponds to previous observations concerning this phage group: The host range of phages Limestone1 (Adriaenssens et al., 2012b), *ϕ*D3 (Czajkowski et al., 2015), XF4, and JA15 (Day et al., 2017) is also limited to *D. solani*.

Phage PP35 also can infect at least one non-virulent *Enterobacteriaceae* isolate associated with soft rot pathogenesis. This strain, F154, yielded a positive signal from the PCR diagnostic test for *Pectobacterium* based on primer set EXPCC (Kang et al., 2003). Further sequencing and bioinformatic analysis of strain F154 genome referred it as a representative of the genus *Lelliottia*. The *Lelliottia* genus was reclassified from *Enterobacter* spp. and divided into two species, *L. nimipressuralis* and *L. amnigena* (previously *Enterobacter nimipressuralis* and *E. amnigenus*, respectively) (Brady et al., 2013), based on genomic MLST analysis and biochemical properties. Two other species of *Lelliottia* were proposed, based on genomic identity and with very slight differences in biochemical properties – *L. jeotgali* (Yuk et al., 2018) and *L. aquatilis* (Kämpfer et al., 2018). *Lelliottia* spp. are environmental bacteria (optimum growth temperature ca. 30°C) that can be isolated from natural water sources, and little is known about their ecological role. They are reportedly associated with food spoilage and phytopathogenesis, including soft rot (Kõiv et al., 2015), and, rarely, with human infections (Kim et al., 2010).

### Infectious Properties of PP35 With Respect to *Dickeya* and *Lelliottia* Strains

**FIGURE 2:**
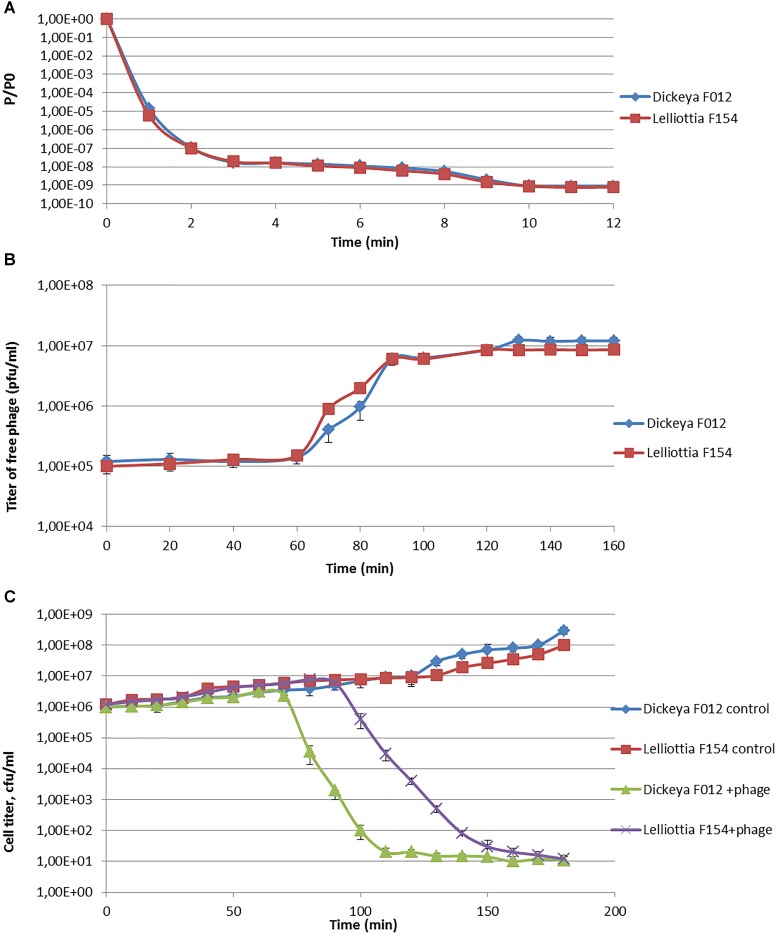
**(A)** Adsorption of phage PP35 on host surface. **(B)** One-step growth curves of PP35 using *D. solani* F012 and *Lelliottia* F154 as hosts. **(C)** Multistep bacterial killing curve in the life cycle of phage PP35. Intact growing *D. solani* F012 and *Lelliottia* F154 cells were used as controls. (MOI = 0.1 in all experiments).

Phage infection parameters were determined with adsorption, one-step-growth, and bacterial elimination assays for phage PP35 on strains *D. solani* F012 and *Lelliottia* sp. F154. Infection kinetics were similar in both cases, with fast (∼ 2 min) and complete adsorption of phages to host bacteria with MOI = 0.1 (Figure 2A). One-step-growth assays also shows similar curves, with a somewhat longer latent period (80 vs. 65 min) and smaller burst size (90 vs. 150 released particles per cell) for F154 (Figure 2B). Within 3 h no substantial secondary growth of bacteria was observed (Figure 2C). Therefore, it is possible to conclude that the lytic infection cycle is realized equally effectively in both bacterial hosts.

### Phage Genome Comparison

**FIGURE 3:**
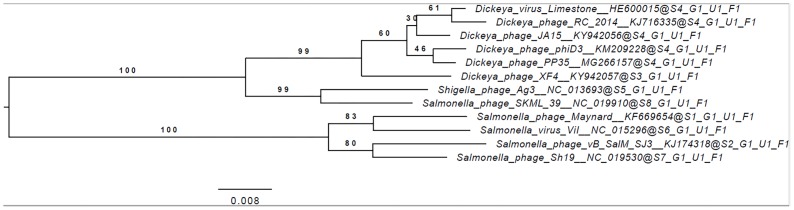
Phylogenomic genome-BLAST distance phylogeny trees inferred using the formula, D4 and yielding average support of 35, 73, and 48%, respectively. The numbers above branches are GBDP pseudo-bootstrap support values from 100 replications.

**FIGURE 4:**
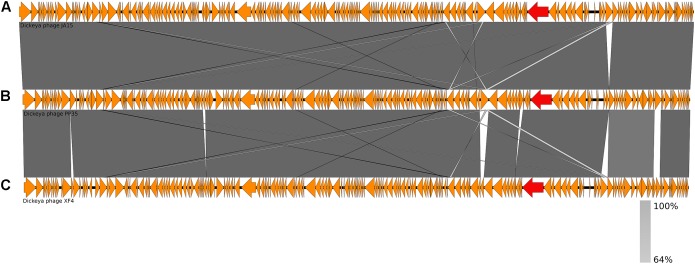
Genome comparison of *Dickeya* phages: **(A)** Limestone, **(B)** PP35, **(C)** *ϕ*D3. Genes encoding tail spike proteins are marked red.

The annotated genome sequence of phage PP35 was deposited in the GenBank database with accession number MG266157.1. Double stranded DNA genome contains 152,048 bp with an average GC content of 49.30%. The genome follows the bidirectional and clustered organization typical for T-even bacteriophages. BLASTN alignment of full-length genomes shows very close relations between PP35 and a number of previously described *Dickeya* phages: Limestone (HE600015.1) (Adriaenssens et al., 2012b), *ϕ*XF4 (KY942057.1), *ϕ*JA15 (KY942056.1) (Day et al., 2017), RC-2014 (KJ716335.1) (Czajkowski et al., 2014b), *ϕ*D3 (Czajkowski et al., 2015). Sequence similarity (Table 2) identified them as belonging to a single species of the genus *Limestonevirus* (Adriaenssens et al., 2017). By transferring annotations from the well described genome and proteome of Limestone, the type phage of the genus (Adriaenssens et al., 2012b), we assigned putative functions to ∼30% of 198 predicted ORFs (Supplementary Table S2). Taxonomic analysis using the VICTOR server (Meier-Kolthoff and Göker, 2017) clusters phage PP35 with the newly proposed family *Ackermannviridae* (Figure 3). Sequences and genome locations of predicted promotors as well as metabolically and structurally essential genes are conserved not only among *Dickeya* -specific Limestone-like phages, but also throughout the genomes of other phages in this family: *Shigella* phage Ag3, *Salmonella* phages SKML-39, Sh19 (Hooton et al., 2011), vB_SalM_SJ3 (Zhang et al., 2014), Vi01 (Pickard et al., 2010), and Maynard (Tatsch et al., 2013) (50–80% pairwise identity) (Table 2). For example, the genome of phage Vi1 was shown to bear hypermodified pyrimidines derived from 5-hydroxymethyl-2’-deoxyuridine (5-hmdU) (Lee et al., 2018). The exact structure of DNA modification in PP35 and the ratio of non-canonical residues are yet to be determined, but the genes responsible for 5-hmdU transformation are conserved in all *Ackermannviridae* phages, and correspond to ORF43 (dUMP hydroxymethyltransferase), ORF44 and ORF183 (kinases), ORF169 (PLP enzyme), ORF113 and ORF187 (alpha-glutamyl/putrescinyl thymidine pyrophosphorylases) in PP35 genome (Supplementary Table S2). Another example of conserved gene clusters is the region located at bp 114,315–128,300 in the PP35 genome. This operon includes 10 ORFs encoding components of the phage baseplate and the adsorption apparatus (Supplementary Table S2). The predicted tail spike sequence encoded by PP35 ORF 156, is identical to the corresponding tail spike sequences of the *Dickeya* phages listed above. The N-terminal domain of ORF156 that is responsible for the attachment of the spike to the phage particle is conserved among all *Ackermannviridae*. Most PP35 genes with unidentified functions are homologous and syntenous with genes in other Limestone-like phages. There are only three unique genes in the PP35 genome, and few indels and duplications (Supplementary Table S2 and Figure 4). Most of these ORFs are hypothetical proteins presumably related to homing endonucleases. No integrases, excisionases, or repressors indicative of lysogenic infection cycle, and no genes encoding toxins or antibiotic resistance mechanisms were detected.

**Table 2:** Genome properties of *Ackermannviridae* phages.

Phage	NCBI #	Genome (kbp)	GC%	ORFs	tRNA	ANI%	Reference
PP35	MG266157.1	152.0	49.3	198	1	100	This work
Limestone	HE600015.1	152.4	49.3	201	1	98.78	Adriaenssens et al., 2012b
*ϕ*D3	KM209228	152.3	49.4	190	1	99.09	Czajkowski et al., 2015
RC2014	KJ716335.1	155.4	49.6	196	1	98.28	Czajkowski et al., 2014b
*ϕ*JA15	KY942056.1	153.8	49.3	198	1	99.16	Day et al., 2017
*ϕ*XF4	KY942057.1	151.5	49.4	195	1	98.90	Day et al., 2017
Ag3	NC_013693.1	158.0	50.4	216	4	87.67	Direct submission
SKML-39	NC_019910.1	159.6	50.2	208	7	87.66	Hooton et al., 2011
Sh19	NC_019530.1	157.8	44.7	166	5	77.45	Hooton et al., 2011
SJ3	KJ174318	162.9	44.4	210	4	77.36	Zhang et al., 2014
Vi01	NC_015296.1	157.7	45.2	208	6	76.45	Pickard et al., 2010
Maynard	KF669654.1	154.7	45.6	200	4	79.71	Tatsch et al., 2013


### Host Genome Comparison

The *D. solani* strain F012 (the PP35 host) genome (NCBI accession numbers PGOJ00000000.1) is similar to other *D. solani* genomes deposited in the public nucleotide databases. IPO2222^T^ is the nearest relative of the *Dickeya* strain F012, with 100% ANI (which is not the same as 100% nucleotide similarity) (Table 3). Homogeneity among *D. solani* strains is thought to reflect the recent evolution of this species (Pritchard et al., 2013). There are only 17 SNPs and InDels between the F012 and IPO2222^T^ genomes. Two putative small CRISPR arrays in contigs PGOJ01000008.1 and PGOJ01000012.1 were identified. Identical CRISPR spacers from those arrays were also found in *Dickeya* spp., *Pantoea vagans*, and *Pectobacterium polaris* genomes.

**Table 3:** Genomic features of Dickeya F012 and type strain IPO2222^T^.

	*Dickeya solani* IPO2222^T^	*Dickeya solani* F012
Genome, bp	4919833	4878843
#genes	4208	4309
CDS	4059	4234
RNA	104	75
tRNA	75	62
ncRNA	7	7
Pseudogenes	45	110


**FIGURE 5:**
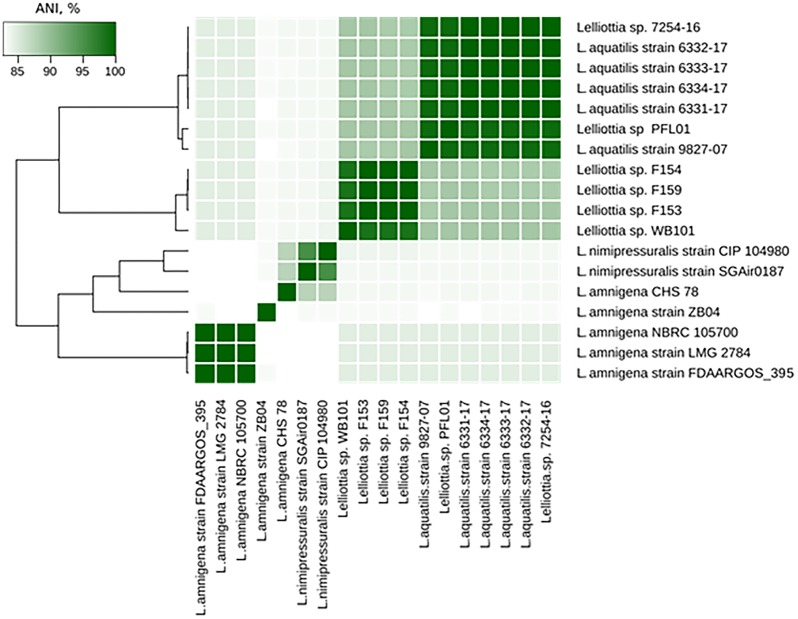
*Lelliottia* genome clusters inferred by ANI between genome assemblies.

The *Lelliottia* strain F154 genome (accession number PKFV00000000.1) is most similar to the draft genome of *Lelliottia sp.* WB101 (GCA_003051885.1) isolated from a denitrifying woodchip bioreactor (98.23% ANI). There is no species designation for F154 and WB101 yet. Genome assemblies of *Lelliottia* strain F153 (draft genome accession number PKFT00000000.1) and F159 (PKFU00000000.1) are included in the same cluster as genomes of F154 and WB101 (ANI > 98.2%). These strains were also isolated in 2014 in the Moscow region, Russia, and are associated with soft rot pathogenesis in potato, however, they are resistant to phage PP35. ANI values between genome assemblies from this cluster and other *Lelliottia* assemblies are not greater than 88.6%, suggesting that these four strains form a distinct species of *Lelliottia* (Figure 5). The newly formed species *L. aquatilis* (which includes *Lelliottia* sp. 7254-16) and *Lelliottia jeotgalii* (PFL01) are most similar to the F154-like cluster, but distinct enough that F154 doesn’t belong to either species (Figure 5). Our findings show the necessity for subsequent phylogenomic research to refine *Lelliottia* strains taxonomy after a thorough study and comparison of their physiological and biochemical properties.

### Properties of the Tail Spike Protein of PP35

Earlier studies suggest that the host range of tailed phages is largely determined by the interaction of their baseplate structures (tail fibers and tail spikes) with molecules on the bacterial surface (Fokine and Rossmann, 2014). Surface polysaccharides, including the *O*-antigens of lipopolysaccharides (LPS), are obvious candidates for receptor interaction with phages (Steinbacher et al., 1996). Tail spikes contain enzymatic domains (Leiman and Molineux, 2008) that depolymerize (Barbirz et al., 2008) or deacetylate (Prokhorov et al., 2017) OPS to allow a phage to attach to the cell surface. Recombinant tail spike proteins of phages infecting *Enterobacteria* (Steinbacher et al., 1996; Barbirz et al., 2008), *Pseudomonas* (Olszak et al., 2017), and *Acinetobacter* (Lee et al., 2017) have been extensively studied functionally and structurally. They were shown to have uniform trimeric β-helical architecture, and enzymatic active sites are located either on the surface of the resulting trimeric prism or within the loops protruding from the prism (Leiman and Molineux, 2008). Recombinant PP35 gp156 tail spike protein forms trimers spontaneously, and can be purified to electrophoretic homogeneity. The protein tends to aggregate at high concentrations, so its crystallization and further detailed structural investigation is impossible at present. In dilute solution (<2 mg/mL) PP35 gp156 is stable for several weeks at 4°C.

### Identification of Surface Polysaccharides of *Dickeya* F012 and *Lelliottia* F154

**FIGURE 6:**
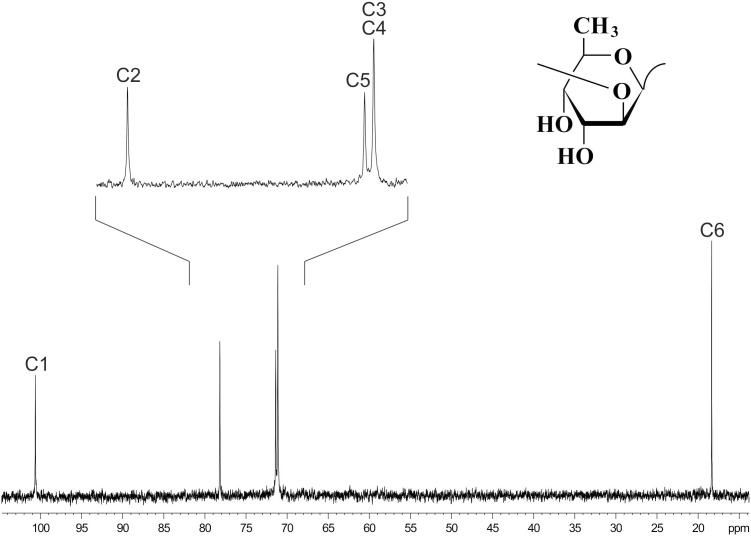
^13^C NMR spectrum of the *O*-polysaccharide of *Dickeya solani* F012. Structure of the *O*-polysaccharide is shown in the inset.

^13^C NMR spectroscopy (Figure 6) showed that the OPS of *D. solani* F012 is composed of 6-deoxy-D-altrose repeating units and is identical to the polysaccharides of five other strains of *D. solani* and *D. dadantii* 3937 reported previously (Ossowska et al., 2017). Analysis of the OPS of *Lelliottia* sp. F154 revealed the same structure with the units →2)- 6-deoxy- β-D-altrose-(1→ (Figure 6). Thus, we hypothesize that the indistinguishable OPS homolog on the surface of the bacterial cells susceptible to phage PP35 is the primary receptor for the PP35 tail spike protein.

**FIGURE 7:**
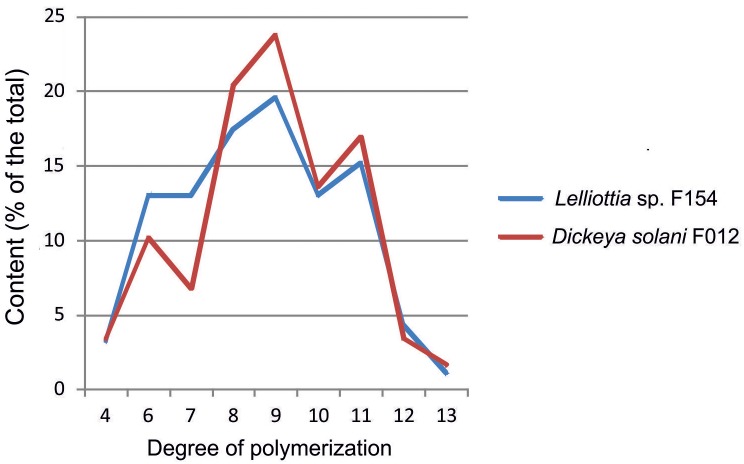
Distribution of oligomers of 6-deoxy-D-altrose derived from the *O*-polysaccharides of *D. solani* F012 and *Lelliottia* sp. F154 by depolymerization with bacteriophage PP35 recombinant tail-spike protein gp156 based on HR ESI MS analysis data.

Depolymerization of the *O*-antigen launches the phage infection process, and the metabolic differences between *Dickeya* and *Lelliottia* do not prevent the infection. To test the hypothesis that PP35 tail spike protein depolymerizes the OPS of strains from these genera, we incubated OPS purified from representative strains with recombinant PP35 gp156. We measured the formation of 6-deoxy-D-altrose oligomers in the range of 4–13 monosaccharide units (degradation products) with high-resolution electrospray ionization mass spectrometry (HR ESI MS). The degradation of *Dickeya* F012 OPS resulted in mostly octa- and nonamers, while the products of *Lelliottia* F154 OPS degradation are distributed more evenly (Figure 7). Therefore, gp156 can hydrolyze 6-deoxy- β-D-altrose-(1→2)-6-deoxy-D-altrose linkage.

We sought the genomic loci responsible for the synthesis and secretion of *O*-antigen. The ORFs involved to lipopolysaccharide synthesis listed in Ossowska et al. (2017) are conserved in *Dickeya* genomes, and most other enterobacterial genomes. They catalyze the formation of lipid A and the linkage between lipid and sugar moieties common for *Enterobacteriaceae* (Knirel and Valvano, 2011). We hypothesized that the ABC transporter-dependent OPS biosynthesis pathway was responsible for *D. solani* OPS production (Greenfield and Whitfield, 2012), because *D. solani* OPS consists of the single repetitive carbohydrate GDP-6-deoxyaltrose. We identified the operon of eight ORFs that fits this model. The annotated ORFs of this operon include: (i) manC (protein accession number WP_022634985.1 in the case of F012 genome), manB (WP_022634986.1), and GDP-mannose 4,6-dehydratase (WP_022634987.1) that isomerizes and activates mannose substrate; (ii) GDP-fucose synthetase (WP_022634990.1) some alleles of which produce GDP-6-deoxyaltrose (Lau and Tanner, 2008); (iii) wzm (WP_026594546.1) permease and wzt (WP_022634989.1) ATP-binding protein that are responsible for the transport of the nascent polysaccharide chain; and (iv) two glycosyltransferases responsible for GDP-6-deoxyaltrose chain initiation, and subsequent polymerization. Homologs of these genes from all *D. solani* genomes present in NCBI GenBank are identical. Distant homologs of some of these ORFs can be found in the genome of *Lelliottia* F154 genome, but they are members of a more complicated cascade of genes. Therefore, we were unable to identify all *Lelliottia* F154 ORFs involved in the 6-deoxy-D-altrose OPS production. Further research is required to identify and characterize those ORFs.

## Discussion

In this work, we characterize the interaction of *Dickeya solani* bacteriophage PP35 with its host. This interaction is believed to be the same as for *sensu stricto* Limestone and *ϕ*D3 phages because they share a host range limited to *D. solani* strains and identical tail spike protein sequences. Attachment of the tail spike to the OPS on the bacterial surface and enzymatic depolymerization of the polysaccharide are key initial events in phage infection. The structure of the OPS is the same for all *D. solani* strains (Ossowska et al., 2016), as well as some other *Pectobacteriaceae* and *Enterobacteriaceae*. We present an example of *Enterobacteriaceae* phage host *Lelliottia* F154. This bacterial strain is evolutionarily distantly related to *Dickeya* sp., shows no virulence to potatoes, and lacks the genetic loci essential for pectate degradation associated with pathogenesis. However, it is infected by phage PP35 effectively, and we’d propose that such a bacterium may be an environmental reservoir for phage PP35.

Investigation of *D. solani* emergence and spread in Europe suggested that the pathogen arose from a single recent evolutionary event (van der Wolf et al., 2014). *D. solani* isolates from different locations in Europe (Belgium, Netherlands, Great Britain, Finland, Israel, Poland, and Russia) that are very closely related according to ANI. Attempts to isolate *D. solani* –specific bacteriophages in samples where the pathogen was detected resulted in the characterization of very similar Myoviruses resembling phage Limestone, the first isolated type representative of the species. Thus, we hypothesize two routes for natural phage infection of the plant pathogen *D. solani*: (i) Phages travel from a single source together with the bacterial population via transfer of seed potatoes, and (ii) a population of bacteriophages propagating on an endemic non- *D. solani* bacterium cross-infects *D. solani.*

There are numerous reports about phages that infect multiple bacterial hosts, especially *Enterobacteriaceae* (Hyman and Abedon, 2010). However, differences in metabolism between environmental and pathogenic bacteria and, especially, the possibility of generalized transduction reported for Limestone-like phages (Day et al., 2017) should lead to greater genetic diversity than is observed (Table 2). On the other hand, Limestone-like phages isolated in nearby locations and using the same *D. solani* strain for enrichment are not identical (Adriaenssens et al., 2012b; Czajkowski et al., 2014a; Day et al., 2017). Phages of this type retain viability on the surface of potato tubers for several weeks (Czajkowski et al., 2017), so the distribution of different phages via seed potato distribution is also possible. Further monitoring and assessment of the diversity of SRP-specific phages is necessary to model the geographic distribution and temporal dynamics of these phages more quantitatively.

The discovery of alternative hosts for SRP-specific phages is useful for phage control applications in addition to its ecological implications. It is desirable to use a well characterized non-pathogenic host for large-scale phage production (Gill and Hyman, 2010). Non-virulent *Lelliottia* strain F154 had similar infection parameters compared to the target pathogenic host and can be a valid choice for industrial propagation of *Limestonevirus.* Limestone-like phages also were shown to be effective for preventive and curative treatment of *D. solani* in field applications (Adriaenssens et al., 2012b). The infection properties of phage PP35 are like those previously described for phages belonging to this species. These properties indicate that it is a promising candidate to include in a phage cocktail for soft rot treatment and prevention.

## Data Availability Statement

The complete genome sequence of phage PP35 has been deposited in the NCBI database under the GenBank accession number MG266157.1. Draft genome assemblies of strains F012 and F154, and related information can be found in the NCBI database under the GenBank Accession numbers PGOJ00000000.1, and PKFV00000000.1, correspondingly.

## Author Contributions

MS, AI, and KAM designed the experiments. APK, MS, AAK, EB, EZ, and EK planned and performed the experiments. AAK, KKM, ST, AI, YK, and KAM performed the data analysis. APK, MS, AI, and KAM wrote the manuscript.

## Conflict of Interest Statement

The authors declare that the research was conducted in the absence of any commercial or financial relationships that could be construed as a potential conflict of interest.

## Footnotes

1 https://github.com/jstjohn/SeqPrep

2 http://rast.nmpdr.org/

3 http://crispr.i2bc.paris-saclay.fr/Server/

4 https://github.com/lmrodriguezr/enveomics

5 http://enve-omics.ce.gatech.edu/ani/index
