# Supplementary material for: Host Specificity of the Dickeya Bacteriophage PP35 Is Directed by a Tail Spike Interaction With Bacterial O-Antigen, Enabling the Infection of Alternative Non-pathogenic Bacterial Host
Source: Front Microbiol. 2019 Jan 11;9:3288. doi: 10.3389/fmicb.2018.03288 (PMC6336734; doi:10.3389/fmicb.2018.03288)
Supplement: Supplementary file 1 [file Table_1.DOCX]

Supplementary Material

Host specificity of the Dickeya bacteriophage PP35 is directed by a tail spike interaction with bacterial O-antigen, and enables the infection of alternative non-pathogenic bacterial host

Anastasia P. Kabanova^1,2^, Mikhail M. Shneider^1^, Aleksei A. Korzhenkov^3^, Eugenia N. Bugaeva^2^, Kirill K. Miroshnikov^4^, Evelina L. Zdorovenko^5^, Eugene E. Kulikov^4^, Stepan V. Toschakov^3,4^, Alexander N. Ignatov^2^, Yuriy A. Knirel^5^, Konstantin A. Miroshnikov^1,2^*

*** Correspondence:**

Dr. Konstantin A. Miroshnikov, Shemyakin-Ovchinnikov Institute of Bioorganic Chemistry, Moscow, Russia, [kmi@ibch.ru](mailto:kmi@ibch.ru)

**Supplementary Table 1.**

| # | Strain designation | Species | Year of isolation | Geographical region | Genus/species determination | PP35 susceptible |
| --- | --- | --- | --- | --- | --- | --- |
| 1 | F012 | Dso | 2010 | Voronezh | NZ_PGOJ00000.1 | + |
| 2 | F043 | Dso | 2010 | Moscow | 16S , PCR | + |
| 3 | F082 | Dso | 1980 | Moscow | PCR | + |
| 4 | F097 | Dso | 2011 | Kaluga | PCR | + |
| 5 | F102 | Dso | 2011 | Moscow | PCR | + |
| 6 | F119 | Dso | 2011 | Tver | PCR | + |
| 7 | F155 | Dso | 2014 | Moscow | PCR | + |
| 8 | F156 | Dso | 2014 | Moscow | 16S, PCR | + |
| 9 | D12 | Dso | 2012 | Moscow | NZ_PGUT00000.1 | + |
| 10 | F069 | Ddi | 2011 | Kashira | 16S, PCR | - |
| 11 | F077 | Ddi | 2002 | Tyumen | 16S, PCR | - |
| 12 | F085 | Ddi | 1979 | Moscow | 16S, PCR | - |
| 13 | F090 | Ddi | 1995 | Kaluga | 16S, PCR | - |
| 14 | SCRI1043 | Pat | ND | Scotland | NC_004547 | - |
| 15 | 21A | Pat | 1990 | Belarus | NZ_CP009125 | - |
| 16 | F004 (PB72) | Pat | 2012 | Moscow | NZ_PDDK00000000.1 | - |
| 17 | F041 | Pat | 2011 | Moscow | 16S, PCR | - |
| 18 | F048 | Pat | 2012 | Tver | 16S, PCR | - |
| 29 | F035 | Ppa | 2012 | Kaluga | 16S, PCR | - |
| 20 | F148 (PB20) | Ppa | 2013 | Moscow | NZ_PDDJ00000000.1 | - |
| 21 | F149 | Ppa | 2013 | Tver | 16S, PCR | - |
| 22 | F126 | Pcb | 2012 | Samara | 16S, PCR | - |
| 23 | F128 | Pcb | 2012 | Samara | 16S, PCR | - |
| 24 | F152 | Pcb | 2014 | Moscow | 16S, PCR | - |
| 25 | F002  (PB69) | Pcc* | 2012 | Moscow | NZ_PDVY00000000.1 | - |
| 26 | F008 | Pcc | 1923 | VKPM | 16S, PCR | - |
| 27 | F018 | Pcc* | 1947 | Moscow | NZ_PDVV00000000.1 | - |
| 28 | F100 | Pcc | 1993 | Tver | 16S, PCR | - |
| 29 | F160 (ATCC15713) | Pcc | ND | Moscow | 16S, MLST  AB242908.1 | - |
| 30 | F131 | Pcc* | 1998 | Kaluga | NZ_PDVW00000000.1 | - |
| 31 | F135 | Pcc* | 1995 | Vladimir | NZ_PDVX00000000.1 | - |
| 32 | F020 | Pcc | 2003 | Moscow | 16S, PCR | - |
| 33 | F053 | Pcc | 2011 | Moscow | 16S, PCR | - |
| 34 | F118 | Pcc | 2005 | Moscow | 16S, PCR | - |
| 35 | F140 | Pcc | 2012 | Moscow | 16S, PCR | - |
| 36 | F150 | Pcc | 2014 | Moscow | 16S, PCR | - |
| 37 | F079 | Stenotropho  monas | 1998 | Voronezh | 16S | - |
| 38 | F096 | Pseudomonas | 1989 | Moscow | 16S | - |
| 39 | F039 | Pseudomonas | 1972 | Lithuania | 16S | - |
| 40 | F153 | Lelliottia | 2014 | Moscow | NZ_PKFT00000000.1 | - |
| 41 | F154 | Lelliottia | 2014 | Moscow | NZ_PKFV00000000.1 | + |
| 42 | F159 | Lelliottia | 2014 | Moscow | NZ_PKFU00000000.1 | - |
| 43 | F084 | Xanthomonas | 2005 | Moscow | 16S | - |
| 44 | F142 | Pantoea | 2013 | Moscow | 16S | - |

+ Denotes plaque formation on the respective host

Pcc* - The strain belongs to the proposed species Pectobacterium maceratum (Shirshikov et al., 2018)

PCR methods used to verify the taxonomy of the isolates were the following: *Pectobacterium carortovorum* subsp*. carotovorum* (Pcc) - (Kang et al., 2003), *Pectobacterium atrosepticum* (Pat) - (De Boer and Ward, 1995), *Pectobacterium carotovorum* subsp. *brasiliense* (Pcb) - (Duarte et al., 2004), *Pectobacterium parmentieri* (former *P.wasabiae*) - (De Boer et al., 2012), *Dickeya solani* (Dso) – conventional PCR adaptation from (van Vaerenbergh et al., 2012), *Dickeya dianthicola* (Ddi) - conventional PCR adaptation from (Pritchard et al., 2013)

**References**

De Boer, S. H., Li, X., and Ward, L. J. (2012). Pectobacterium spp. Associated with Bacterial Stem Rot Syndrome of Potato in Canada. *Phytopathology* 102, 937–947. doi:10.1094/PHYTO-04-12-0083-R.

De Boer, S. H., and Ward, L. J. (1995). PCR detection of Erwinia carotovora subsp atroseptica associated with potato tissue. *Phytopathology* 85, 854–858. doi:10.1094/Phyto-85-854.

Duarte, V., De Boer, S. H., Ward, L. J., and De Oliveira, A. M. R. (2004). Characterization of atypical Erwinia carotovora strains causing blackleg of potato in Brazil. *Journal of Applied Microbiology* 96, 535–545. doi:10.1111/j.1365-2672.2004.02173.x.

Kang, H. W., Kwon, S. W., and Go, S. J. (2003). PCR-based specific and sensitive detection of Pectobacterium carotovorum ssp. carotovorum by primers generated from a URP-PCR fingerprinting-derived polymorphic band. *Plant Pathology* 52, 127–133. doi:10.1046/j.1365-3059.2003.00822.x.

Pritchard, L., Humphris, S., Saddler, G. S., Parkinson, N. M., Bertrand, V., Elphinstone, J. G., et al. (2013). Detection of phytopathogens of the genus Dickeya using a PCR primer prediction pipeline for draft bacterial genome sequences. *Plant Pathology* 62, 587–596. doi:10.1111/j.1365-3059.2012.02678.x.

Shirshikov, F. V., Korzhenkov, A. A., Miroshnikov, K. K., Kabanova, A. P., Barannik, A. P., Ignatov, A. N., et al. (2018). Draft Genome Sequences of New Genomospecies “ *Candidatus* Pectobacterium maceratum” Strains, Which Cause Soft Rot in Plants. *Genome Announcements* 6, e00260-18. doi:10.1128/genomeA.00260-18.

van Vaerenbergh, J., Baeyen, S., de Vos, P., and Maes, M. (2012). Sequence diversity in the Dickeya flic gene: Phylogeny of the Dickeya genus and taqman® PCR for “D. solani”, new biovar 3 variant on potato in Europe. *PLoS ONE* 7. doi:10.1371/journal.pone.0035738.
