# Supplementary material for: Host Specificity of the Dickeya Bacteriophage PP35 Is Directed by a Tail Spike Interaction With Bacterial O-Antigen, Enabling the Infection of Alternative Non-pathogenic Bacterial Host
Source: Front Microbiol. 2019 Jan 11;9:3288. doi: 10.3389/fmicb.2018.03288 (PMC6336734; doi:10.3389/fmicb.2018.03288)
Supplement: Supplementary file 2 [file Table_2.DOCX]

Supplementary Material

Host specificity of the Dickeya bacteriophage PP35 is directed by a tail spike interaction with bacterial O-antigen, and enables the infection of alternative non-pathogenic bacterial host

Anastasia P. Kabanova^1,2^, Mikhail M. Shneider^1^, Aleksei A. Korzhenkov^3^, Eugenia N. Bugaeva^2^, Kirill K. Miroshnikov^4^, Evelina L. Zdorovenko^5^, Eugene E. Kulikov^4^, Stepan V. Toschakov^3,4^, Alexander N. Ignatov^2^, Yuriy A. Knirel^5^, Konstantin A. Miroshnikov^1,2^*

**Supplementary Figures and Tables**

**Supplementary Table 2.**

Putative gene functions of *Dickeya solani* phage РР35 (vB_DsoM_PP35), 152 048 bp genome, 198 ORFs.

| ORF | Position in genome, bp | Translation, aa | Homolog in Limestone | Closest relative | Query coverage (%)-identity (%)-E value | Predicted function |
| --- | --- | --- | --- | --- | --- | --- |
| 01 | 1..2757 | 918 | ORF001 | RIIA protein [Dickeya phage XF4] ASD51387.1 | 100-99-0 | RIIA protein, premature lysis protection |
| 02 | 2789..4351 | 520 | ORF001 | RIIB protein [Dickeya phage XF4] ASD51388.1 | 100-98-0 | RIIB protein, lysis inhibitor |
| 03 | 4403..4708 | 101 | ORF003 | hypothetical protein G379_gp201 [Dickeya virus Limestone] | 100-100-5e-70 | hypothetical protein, factor independent terminator |
| 04 | 4677..5102 | 141 | ORF004 | hypothetical protein [Shigella phage Ag3] YP_003358491.1 | 100-91-5e-78 | putative transcription regulation protein |
| 05 | 5133..5525 | 130 | ORF005 | hypothetical protein G379_gp199 [Dickeya virus Limestone] YP_007237327.1 | 100-100-4e-91 | hypothetical protein |
| 06 | 5504..6313 | 269 | ORF006 | unknown structural protein [Dickeya virus Limestone] YP_007237328.1 | 100-100-0 | putative hoc protein * also referred as tail fiber or tail spike protein for Ag3, SKML-39, Vi1 |
| 07 | 6316..6549 | 77 | ORF007 | hypothetical protein HQ80_0012 [Dickeya phage phiD3] AIM51365.1 | 100-100-2e-48 | hypothetical protein |
| 08 | 6638..7096 | 152 | ORF008 | putative histone-like protein [Dickeya phage phiD3] AIM51334.1 | 100-100-5e-110 | hypothetical protein |
| 09 | 7126..7452 | 108 | ORF009 | hypothetical protein [Dickeya phage XF4] ASD51395.1 | 100-100-1e-73 | hypothetical protein, putative DUF1768 protein |
| 10 | 7449..8030 | 193 | ORF011 | hypothetical protein HQ80_0015 [Dickeya phage phiD3] AIM51277.1 | 100-100-4e-143 | putative pyrophosphatase |
| 11 | 8027..8857 | 276 | ORF012 | putative HNH homing endonuclease domain protein [Dickeya phage phiD3] ATW62017.1 | 100-98-0 | putative HNH endonuclease |
| 12 | 8850..10751 | 633 | ORF013 | putative DNA topoisomerase II [Dickeya phage XF4] ASD51398.1 | 100-98-0 | putative DNA topoisomerase II |
| 13 | 10753..12084 | 443 | ORF015 | DNA gyrase/topoisomerase IV subunit A family protein [Dickeya phage phiD3] AIM51433.1 | 100-100-0 | putative DNA topoisomerase II |
| 14 | 12127..12414 | 95 | ORF016 | hypothetical protein G379_gp189 [Dickeya virus Limestone] YP_007237338.1 | 100-100-1e-60 | hypothetical protein, membrane protein |
| 15 | 12674..12760 | 28 | no | hypothetical protein DA66_0185 [Dickeya phage RC-2014] YP_009103024.1 | 100-96-2e-19 | hypothetical protein |
| 16 | 12831..13145 | 102 | ORF018 | hypothetical protein G379_gp187 [Dickeya virus Limestone] YP_007237340.1 | 100-100-2e-68 | hypothetical protein |
| 17 | 13145..13651 | 168 | ORF019 | putative deoxycytidylate deaminase [Dickeya virus Limestone] YP_007237341.1 | 100-100-2e-121 | putative deoxycytidylate deaminase |
| 18 | 13662..14069 | 135 | ORF020 | hypothetical protein [Salmonella phage SKML-39] YP_007236238.1 | 100-99-2e-91 | putative membrane protein |
| 19 | complement (14281..14895) | 204 | ORF021 | putative head completion protein gp4 [Dickeya virus Limestone] YP_007237343.1 | 100-100-6e-150 | putative endonuclease |
| 20 | complement (14895..15632) | 245 | ORF022 | putative endonuclease segB domain protein [Dickeya phage phiD3] AIM51404.1 | 100-100-0 | putative HNH homing endonuclease |
| 21 | 15683..16651 | 322 | ORF023 | putative tail tube associated baseplate protein gp48 [Dickeya virus Limestone] YP_007237345.1 | 100-100-0 | tail tube initiation protein |
| 22 | 16696..18339 | 547 | ORF024 | putative homing endonuclease F-LimIII [Dickeya virus Limestone] YP_007237346.1 | 100-100-0 | putative homing endonuclease |
| 23 | 18342..18896 | 184 | ORF025 | putative baseplate wedge subunit gp53 [Dickeya virus Limestone] YP_007237347.1 | 100-100-7e-134 | baseplate structural protein, wedge subunit |
| 24 | 18893..20281 | 462 | ORF026 | putative baseplate hub subunit gp27 [Dickeya virus Limestone] YP_007237348.1 | 100-99-0 | baseplate hub component |
| 25 | 20292..22241 | 649 | ORF027 | putative tail length tape measure protein [Dickeya virus Limestone] YP_007237349.1 | 100-99-0 | tape measure protein |
| 26 | complement (22242..22901) | 219 | ORF028 | putative loader of gp41 DNA helicase gp59 [Dickeya virus Limestone] YP_007237350.1 | 100-100-5e-163 | loader of DNA helicase |
| 27 | complement (22895..23149) | 84 | ORF029 | hypothetical protein [Dickeya virus Limestone] P_007237351.1 | 100-100-1e-54 | putative transcription regulator |
| 28 | complement (23151..23375) | 74 | ORF030 | hypothetical protein [Dickeya virus Limestone] YP_007237352.1 | 100-100-9e-46 | hypothetical protein |
| 29 | complement (23405..23602) | 65 | ORF031 (longer in PP35) | hypothetical protein [Salmonella phage SKML-39] YP_007236229.1 | 95-77-5e-25 | hypothetical protein |
| 30 | complement (23602..25026) | 474 | ORF032 | putative DNA ligase gp30 [Dickeya virus Limestone] YP_007237354.1 | 100-100-0 | putative DNA ligase |
| 31 | complement (25169..25390) | 73 | ORF033 | hypothetical protein [Dickeya virus Limestone] YP_007237355.1 | 100-100-1e-46 | hypothetical protein |
| 32 | complement (25390..25506) | 38 | ORF034 | hypothetical protein [Dickeya virus Limestone] YP_007237356.1 | 100-100-6e-17 | hypothetical protein |
| 33 | complement (25503..26699) | 398 | ORF035 | hypothetical protein [Dickeya phage JA15] ASD51219.1 | 100-93-0 | hypothetical protein |
| 34 | complement (26827..27429) | 200 | ORF036 | putative homing endonuclease F-LimIV [Dickeya virus Limestone] YP_007237358.1 | 100-100-5e-149 | putative homing endonuclease |
| 35 | complement( 27459..27755) | 98 | ORF037 | hypothetical protein [Dickeya virus Limestone] YP_007237359.1 | 100-100-6e-66 | putative transcription regulator |
| 36 | complement (27771..28094) | 107 | ORF038 | hypothetical protein [Dickeya virus Limestone] YP_007237360.1 | 100-99-1e-73 | hypothetical protein |
| 37 | complement (28156..29583) | 475 | ORF039 | putative DNA primase-helicase subunit gp41 [Dickeya virus Limestone] YP_007237361.1 | 100-100-0 | putative DNA primase-helicase subunit |
| 38 | complement (29591..29920) | 109 | ORF040 | hypothetical protein HQ80_0038 [Dickeya phage phiD3] AIM51443.1 | 100-100-5e-73 | hypothetical protein |
| 39 | complement( 29898..30968) | 356 | ORF041 | recA bacterial DNA recombination family protein [Dickeya phage phiD3] AIM51312.1 | 100-100-0 | RecA-like recombination protein, UvsX-like protein |
| 40 | complement (30968..31510) | 180 | ORF042 | hypothetical protein [Dickeya virus Limestone] YP_007237364.1 | 100-100-4e-131 | hypothetical protein |
| 41 | complement (31510..32064) | 184 | ORF043 | dUTPase family protein [Dickeya phage RC-2014] YP_009102850.1 | 100-100-9e-136 | dUTPase family protein |
| 42 | complement (32061..32630) | 189 | ORF044 | putative dNMP kinase gp1 [Dickeya virus Limestone] YP_007237366.1 | 100-100-1e-138 | deoxynucleotide monophosphate kinase |
| 43 | complement (32627..33673) | 348 | ORF045 | putative thymidylate synthase [Dickeya virus Limestone] YP_007237367.1 | 100-100-0 | putative thymidylate synthase |
| 44 | complement (33690..34412) | 240 | ORF046 | putative thymidylate kinase [Dickeya virus Limestone] YP_007237368.1 (other homologs are C-terminally truncated) | 100-100-9e-179 | putative thymidylate kinase |
| 45 | complement (34487..35413) | 308 | ORF047 | hypothetical protein [Dickeya virus Limestone] YP_007237369.1 | 100-100-0 | hypothetical protein |
| 46 | complement (35619..36371) | 250 | ORF048 | hypothetical protein [Dickeya virus Limestone] YP_007237370.1 | 100-100-0 | hypothetical protein |
| 47 | complement (36438..37139) | 233 | ORF049 | putative DNA end protector protein [Dickeya phage phiD3] AIM51401.1 | 100-100-1e-173 | putative DNA end protector protein |
| 48 | 37194..38138 | 314 | ORF050 | putative baseplate tail tube initiator gp54 [Dickeya virus Limestone] YP_007237372.1 | 100-100-0 | tail tube initiator |
| 49 | complement (38165..39211) | 348 | ORF051 | putative ssDNA binding protein gp32 [Dickeya virus Limestone] YP_007237373.1 | 100-100-0 | putative ssDNA binding protein |
| 50 | complement (39310..39549) | 79 | ORF052 | hypothetical protein [Dickeya virus Limestone] YP_007237374.1 | 100-100-2e-52 | hypothetical protein |
| 51 | complement (39557..39802) | 81 | ORF053 | putative late promoter transcription accessory protein gp33 [Dickeya virus Limestone] YP_007237375.1 | 100-100-1e-48 | late promoter transcription accessory protein |
| 52 | complement (39795..40040) | 81 | ORF054 | putative regulatory protein [Dickeya virus Limestone] YP_007237376.1 | 100-100-8e-53 | putative FmdB family regulatory protein |
| 53 | complement (40027..40338) | 103 | ORF055 | hypothetical protein [Dickeya virus Limestone] YP_007237377.1 | 100-100-1e-66 | hypothetical protein w/transmembrane domains |
| 54 | complement (40338..40928) | 196 | ORF056 | putative holliday junction resolvase [Dickeya phage XF4] ASD51439.1 | 100-100-9e-147 | putative holliday junction resolvase |
| 55 | complement (40980..41549) | 189 | ORF014 | hypothetical protein [Salmonella phage SKML-39] YP_007236253.1 | 98-48-2e-49 | putative HNH endonuclease |
| 56 | complement (41599..42090) | 163 | ORF057 | hypothetical protein [Dickeya phage XF4] ASD51440.1 | 100-100-3e-116 | hypothetical protein |
| 57 | complement (42068..42592) | 174 | ORF058 | hypothetical protein [Dickeya virus Limestone] YP_007237380.1 | 100-100-2e-127 | hypothetical protein |
| 58 | 42643..43449 | 268 | ORF59 | base plate family protein [Dickeya phage RC-2014] YP_009102866.1 | 100-100-0 | baseplate assembly chaperone protein |
| 59 | 43960..45570 | 536 | ORF061 | putative baseplate hub subunit & tail lysozyme gp5 [Dickeya virus Limestone] YP_007237383.1 | 100-100-0 | baseplate hub protein |
| 60 | 45645..46025 | 126 | ORF062 | putative baseplate wedge subunit [Dickeya phage JA15] ASD51246.1 | 100-100-3e-87 | baseplate wedge protein |
| 61 | complement (46026..46415) | 129 | ORF063 | hypothetical protein [Dickeya phage JA15] ASD51247.1 | 100-100-7e-91 | hypothetical protein |
| 62 | complement (46422..46883) | 153 | ORF064 | hypothetical protein [Dickeya phage JA15] ASD51248.1 | 100-100-1e-110 | hypothetical protein |
| 63 | complement (46966..47766) | 266 | ORF012 (30% similarity) | putative homing endonuclease F-LimI [Dickeya phage JA15] ASD51249.1 | 100-100-0 | putative homing endonuclease |
| 64 | complement (47814..48038) | 74 | ORF065 | putative glutaredoxin [Dickeya phage XF4] ASD51449.1 | 100-99-3e-47 | putative glutaredoxin |
| 65 | complement (48048..49154) | 368 | ORF066 | putative ribonucleoside-diphosphate reductase subunit beta [Dickeya phage JA15] ASD51251.1 | 100-100-0 | putative ribonucleoside-diphosphate reductase beta subunit |
| 66 | complement (49213..49575) | 120 | ORF012 (39% similarity) | homing endonuclease [Klebsiella phage May] AUG88058.1 | 99-47-8e-27 | putative homing endonucleas |
| 67 | complement (49575..52877) | 1100 | ORF068 | putative ribonucleoside-diphosphate reductase subunit alpha [Dickeya phage JA15] ASD51253.1 | 100-100-0 | putative ribonucleoside-diphosphate reductase subunit alpha |
| 68 | complement (52961..53803) | 280 | ORF069 | putative PhoH-like phosphate starvation-inducible protein [Dickeya virus Limestone] YP_007237391.1 | 100-100-0 | putative PhoH-like phosphate starvation-inducible protein |
| 69 | complement (53908..54702) | 264 | ORF070 | putative peptidoglycan binding domain protein [Dickeya phage phiD3] AIM51349.1 | 100-100-0 | putative N-acetylmuramidase |
| 70 | complement (54770..55075) | 101 | ORF071 | hypothetical protein [Dickeya virus Limestone] YP_007237393.1 | 100-100-2e-69 | hypothetical protein |
| 71 | complement (55072..55260) | 62 | no y | hypothetical protein [Shigella phage Ag3] YP_003358575.1 | 100-100-1e-37 | hypothetical protein |
| 72 | complement (55322..55810) | 162 | ORF073 | hypothetical protein [Dickeya virus Limestone] YP_007237395.1 | 100-100-3e-111 | hypothetical protein |
| 73 | complement (56006..56212) | 68 | ORF074 | hypothetical protein HQ80_0073 [Dickeya phage phiD3] AIM51393.1 | 100-100-4e-43 | hypothetical protein |
| 74 | complement (56209..57066) | 285 | ORF075 | putative DNA primase subunit [Dickeya phage phiD3] AIM51461.1 | 100-100-0 | putative DNA primase subunit |
| 75 | complement (57264..57920) | 218 | ORF036, ORF076 (less conserved) | putative homing endonuclease F-LimV [Dickeya virus Limestone] YP_007237398.1 | 100-100-1e-162 | putative homing endonuclease |
| 76 | complement (57920..58534) | 204 | ORF077 | hypothetical protein [Dickeya virus Limestone] YP_007237399.1 | 100-100-1e-173 | hypothetical protein |
| 77 | complement (58592..58945) | 117 | ORF078 | hypothetical protein [Dickeya virus Limestone] YP_007237400.1 | 100-100-3e-81 | putative starvation-induced DNA protecting protein |
| 78 | complement (58996..59235)- | 79 | ORF079 | hypothetical protein [Dickeya virus Limestone] YP_007237401.1 | 100-100-2e-50 | hypothetical protein |
| 79 | complement (59245..59814) | 189 | ORF080 | unknown structural protein [Dickeya virus Limestone] YP_007237402.1 | 100-100-6е-137 | hypothetical protein |
| 80 | complement (59871..61466) | 531 | ORF081 (w/gaps) | hypothetical protein DA66_0048 [Dickeya phage RC-2014] YP_009102888.1 | 100-100-0 | hypothetical protein structural |
| 81 | complement (61511..61738) | 75 | ORF083 | hypothetical protein [Dickeya virus Limestone] YP_007237405.1 | 100-100-2e-47 | hypothetical protein |
| 82 | complement (61782..62117) | 111 | ORF084 | hypothetical protein [Dickeya virus Limestone] YP_007237406.1 | 100-100-1e-76 | hypothetical protein |
| 83 | complement (62224..62862) | 212 | ORF085 | unknown structural protein [Dickeya virus Limestone] YP_007237407.1 | 100-100-1e-172 | hypothetical protein  structural |
| 84 | complement (62862..63476) | 204 | ORF086 | putative RegB protein [Dickeya virus Limestone] YP_007237408.1 | 100-100-3e-151 | hypothetical protein |
| 85 | complement (63576..63860) | 94 | ORF087 | hypothetical protein [Dickeya virus Limestone] YP_007237409.1 | 100-100-7e-65 | hypothetical protein |
| 86 | complement (64114..66450) | 778 | ORF088 | recF/RecN/SMC N terminal domain protein [Dickeya phage phiD3] | 100-100-0 | putative recombination endonuclease subunit |
| 87 | complement (66453..67568) | 371 | ORF089 | putative endonuclease gp47 [Dickeya virus Limestone] YP_007237412.1 | 100-100-0 | putative endonuclease |
| 88 | complement (67568..68299) | 243 | ORF091 | putative sigma factor late transcription [Dickeya phage JA15] ASD51274.1 | 100-100-0 | putative sigma factor late transcription |
| 89 | complement (68287..69021) | 244 | ORF076 | putative homing endonuclease F-LimVI [Dickeya phage JA15] ASD51275.1 | 100-100-1e-79 | putative homing endonuclease |
| 90 | complement (69030..69557) | 175 | ORF092 | putative ribonuclease HI [Dickeya virus Limestone] YP_007237414.1 | 100-100-3e-126 | putative ribonuclease H |
| 91 | 69603..70370 | 255 | ORF093 | unknown structural protein [Dickeya virus Limestone] YP_007237415.1 | 100-100-0 | hypothetical protein |
| 92 | complement (70367..72085) | 572 | ORF094 | putative ATP-dependent DNA helicase [Dickeya virus Limestone] YP_007237416.1 | 100-100-0 | putative ATP-dependent DNA helicase |
| 93 | complement (72215..72493) | 92 | ORF095 | putative DNA binding protein [Dickeya virus Limestone] YP_007237417.1 | 100-100-3e-57 | putative DNA-binding protein |
| 94 | complement (72584..72862) | 92 | ORF096 | hypothetical protein [Dickeya virus Limestone] YP_007237418.1 | 100-100-2e-61 | hypothetical protein |
| 95 | complement (72864..73016) | 50 | ORF097 | hypothetical protein [Dickeya virus Limestone] YP_007237419.1 | 100-100-3e-28 | hypothetical protein |
| 96 | complement (73075..73905) | 276 | ORF098 | hypothetical protein [Dickeya virus Limestone] YP_007237420.1 | 100-100-0 | hypothetical protein |
| 97 | complement (73955..74287) | 110 | ORF099 | hypothetical protein [Dickeya phage JA15] ASD51283.1 | 100-100-3e-76 | putative o-spanin |
| 98 | complement (74284..74970) | 228 | ORF100 | hypothetical protein [Dickeya virus Limestone] | 100-100-6e-128 | putative i-spanin |
| 99 | complement (75055..75504) | 149 | ORF101 | unknown structural protein [Dickeya virus Limestone] YP_007237423.1 | 100-100-9e-106 | hypothetical protein |
| 100 | complement (75527..75769) | 80 | ORF102 | hypothetical protein [Dickeya virus Limestone] YP_007237424.1 | 100-100-9e-51 | hypothetical protein |
| 101 | complement (75772..76059) | 95 | ORF103 (N-term truncated | hypothetical protein DA66_0067 [Dickeya phage RC-2014] YP_009102907.1 | 100-100-8e-60 | hypothetical protein |
| 102 | complement (76056..76430) | 124 | ORF104 | hypothetical protein [Dickeya virus Limestone] | 100-100-9e-86 | hypothetical protein |
| 103 | complement (76443..76898) | 151 | ORF105 | putative pyimidine dimer DNA glycosylase DenV [Dickeya virus Limestone] YP_007237427.1 | 100-100-1e-106 | putative pyimidine dimer DNA glycosylase |
| 104 | complement (76959..77198) | 79 | ORF106 | hypothetical protein [Dickeya virus Limestone] YP_007237428.1 | 100-100-3e-48 | hypothetical protein |
| 105 | complement (77322..77645) | 107 | ORF107 | putative acyl carrier protein [Dickeya virus Limestone] YP_007237429.1 | 100-100-5e-70 | putative acyl carrier protein |
| 106 | complement (77688..79892) | 734 | ORF108 | putative vWa containing protein [Dickeya virus Limestone] | 100-99-0 | von Willebrand factor type A domain protein |
| 107 | complement (79885..80427) | 180 | ORF109 | hypothetical protein [Dickeya phage JA15] ASD51293.1 | 100-100-1e-130 | hypothetical protein |
| 108 | complement (80470..80802) | 110 | ORF110 | utative nicotinamide phosphoribosyltransferase NadV [Dickeya virus Limestone] YP_007237432.1 | 100-100-1e-74 | putative nicotinate phosphoribosyltransferase |
| 109 | complement (80902..81093) | 63 | ORF110 | hypothetical protein HQ80_0107 [Dickeya phage phiD3] AIM51453.1 | 100-100-4e-39 | hypothetical protein |
| 110 | complement (81142..81486) | 114 | No homology | hypothetical protein HQ80_0108 [Dickeya phage phiD3] AIM51352.1 | 100-100-3e-80 | hypothetical protein |
| 111 | complement (81638..81919) | 93 | ORF108 (low similarity) | hypothetical protein [Dickeya phage JA15] ASD51297.1 | 100-100-7e-64 | hypothetical protein |
| 112 | complement (82035..82820) | 261 | ORF114 | putative homing endonuclease F-LimVI [Dickeya phage JA15] ASD51298.1 | 100-100-0 | putative homing endonuclease |
| 113 | complement (82822..84012) | 396 | ORF115 | hypothetical protein [Dickeya phage JA15] ASD51299.1 | 100-100-0 | hypothetical protein |
| 114 | complement (84081..84947) | 288 | ORF116 | hypothetical protein HQ80_0113 [Dickeya phage phiD3] AIM51412.1 | 100-100-0 | putative NTP pyrophosphohydrolase |
| 115 | complement (84964..85428) | 154 | ORF117 | translation repressor domain protein [Dickeya phage phiD3] AIM51415.1 | 100-100-9e-112 | translational repressor protein |
| 116 | complement (85458..85880) | 140 | ORF118 | putative clamp loader subunit DNA polymerase accessory protein [Dickeya phage phiD3] AIM51321.1 | 100-100-5e-99 | putative clamp loader subunit |
| 117 | complement (85885..86874) | 329 | ORF119 | ATPase [Dickeya phage phiD3] AIM51451.1 | 100-100-0 | putative clamp loader subunit DNA polymerase accessory protein |
| 118 | complement (86954..87622) | 222 | ORF120 | sliding clamp C terminal family protein [Dickeya phage phiD3] AIM51336.1 | 100-100-3e-162 | putative sliding clamp |
| 119 | 87967..88344 | 125 | ORF121 | hypothetical protein [Dickeya virus Limestone] YP_007237443.1 | 100-99-1e-84 | hypothetical protein |
| 120 | complement (88335..88664) | 109 | ORF122 | putative UvsW protein [Dickeya virus Limestone] YP_007237444.1 | 100-100-1e-75 | putative UvsW protein |
| 121 | complement (88661..89605) | 314 | ORF123 | putative intron I-LimI [Dickeya virus Limestone] YP_007237445.1 | 95-99-0 | homing endonuclease |
| 122 | complement (89538..90929) | 463 | ORF124 most homologs C-terminally truncated | DEAD/DEAH box helicase family protein [Dickeya phage phiD3] AIM51410.1 | 100-100-0 | putative UvsW protein |
| 123 | complement (90958..91704) | 248 | ORF125 | putative exonuclease [Dickeya virus Limestone] YP_007237447.1 | 100-100-0 | putative exonuclease |
| 124 | complement (91704..92159) | 151 | ORF126 | putative UvsY protein [Dickeya virus Limestone] YP_007237448.1 | 100-100-5e-107 | putative UvsY protein |
| 125 | complement (92202..92702) | 166 | ORF127 | putative tail completion & sheath stabilizer protein gp3 [Dickeya virus Limestone] YP_007237449.1 | 100-100-1e-115 | putative tail completion protein |
| 126 | 92731..93381 | 216 | ORF128 | hypothetical protein [Dickeya virus Limestone] YP_007237450.1 | 100-100-5e-157 | hypothetical protein |
| 127 | complement (93382..94110) | 242 | ORF129 | unknown structural protein [Dickeya virus Limestone] YP_007237451.1 | 100-100-1e-157 | hypothetical protein |
| 128 | complement (94148..94315) | 55 | ORF130 | hypothetical protein [Dickeya virus Limestone] YP_007237452.1 | 100-100-3e-32 | hypothetical protein |
| 129 | complement (94356..94778) | 140 | ORF131 | hypothetical protein [Dickeya virus Limestone] YP_007237453.1 | 100-100-7e-99 | hypothetical protein |
| 130 | complement (94784..95104) | 106 | ORF132 | hypothetical protein [Dickeya virus Limestone] YP_007237454.1 | 100-100-3e-70 | hypothetical protein |
| 131 | complement (95239..95445) | 68 | ORF133 | hypothetical protein [Dickeya virus Limestone] YP_007237455.1 | 100-93-3e-38 | hypothetical protein |
| 132 | complement (95549..95710) | 53 | ORF134 | hypothetical protein [Dickeya virus Limestone] YP_007237456.1 | 100-98-1e-30 | hypothetical protein |
| 133 | complement (95793..96233) | 146 | ORF135 | hypothetical protein [Shigella phage Ag3] YP_003358640.1 | 100-99-2e-103 | hypothetical protein |
| 134 | complement (96388..96561) | 57 | ORF136 | hypothetical protein [Dickeya virus Limestone] YP_007237458.1 | 100-100-1e-33 | hypothetical protein |
| 135 | complement (96658..98133) | 491 | ORF137 | putative homing endonuclease F-LimVII [Dickeya virus Limestone] YP_007237459.1 | 100-100-0 | putative homing endonuclease |
| 136 | complement (98241..99563) | 440 | ORF138 | putative major capsid protein gp23 [Dickeya virus Limestone] YP_007237460.1 | 100-100-0 | putative major capsid protein |
| 137 | complement (99655..100527) | 290 | ORF139 | putative scaffolding protein gp22 [Dickeya virus Limestone] YP_007237461.1 | 100-100-0 | putative scaffolding protein |
| 138 | complement (100573..  101238) | 221 | ORF140 | putative prohead protease gp21 [Dickeya virus Limestone] YP_007237462.1 | 100-100-6e-163 | putative prohead protease |
| 139 | complement  (101249..  101554) | 101 | ORF141 | putative prohead core protein [Dickeya virus Limestone] YP_007237463.1 | 100-100-1e-64 | putative prohead core protein |
| 140 | complement (101565.  .101732) | 55 | ORF142 | hypothetical protein [Dickeya virus Limestone] YP_007237464.1 | 100-100-3e-30 | hypothetical protein |
| 141 | complement (101771..  103462) | 563 | ORF143 | putative portal protein [Dickeya virus Limestone] YP_007237465.1 | 100-99-0 | putative portal protein |
| 142 | complement (103530..  104063) | 177 | ORF144 | putative tail tube protein [Dickeya phage XF4] ASD51525.1 | 100-98-6e-126 | putative tail tube protein |
| 143 | 104354..106144 | 596 | ORF145 | putative homing endonuclease F-LimVIII [Dickeya virus Limestone] YP_007237467.1 | 100-100-0 | putative homing endonuclease |
| 144 | complement (106171..  108069) | 632 | ORF146 | putative tail sheath protein gp18 [Dickeya virus Limestone] YP_007237468.1 | 100-100-0 | putative tail sheath protein |
| 145 | complement (108122..  110329) | 735 | ORF147 | pretoxin HINT domain protein [Dickeya phage phiD3] AIM51424.1 | 100-99-0 | putative terminase large subunit |
| 146 | complement (110319..  111197) | 292 | ORF148 | NUMOD3 motif family protein [Dickeya phage phiD3] AIM51421.1 | 100-100-0 | putative homing endonuclease |
| 147 | complement (111175..  111876) | 233 | ORF149 | putative terminase small subunit gp16 [Dickeya virus Limestone] YP_007237471.1 | 100-99-1e-162 | putative terminase small subunit |
| 148 | complement (111879..  112574) | 231 | ORF150 | putative tail sheath stabilizer gp15 [Dickeya virus Limestone] YP_007237472.1 | 100-100-4e-174 | putative tail sheath stabilizer |
| 149 | complement(112577..  113227) | 216 | ORF151 | putative neck protein gp14 [Dickeya virus Limestone] YP_007237473.1 | 100-100-3e-159 | putative neck protein |
| 150 | 113286..113993 | 235 | ORF152 | hypothetical protein [Dickeya virus Limestone] YP_007237474.1 | 100-100-4e-173 | hypothetical protein |
| 151 | 114062..114286 | 74 | ORF153 | hypothetical protein [Dickeya virus Limestone] YP_007237475.1 | 100-100-8e-46 | hypothetical protein |
| 152 | complement (114315..  115067) | 250 | ORF154 | putative neck protein gp13 [Dickeya virus Limestone] YP_007237476.1 | 100-100-0 | putative neck protein |
| 153 | complement (115057..  115395) | 112 | ORF155 | hypothetical protein [Dickeya virus Limestone] YP_007237477.1 | 100-100-2e-87 | hypothetical protein |
| 154 | complement (115376..  115627) | 83 | ORF156 | hypothetical protein [Dickeya virus Limestone] YP_007237478.1 | 100-100-3e-53 | hypothetical protein |
| 155 | complement (115678..  120516) | 1612 | ORF157 | hypothetical protein HQ80_0154 [Dickeya phage phiD3] AIM51422.1 | 100-100-0 | baseplate protein vrlC, family DUF4815 |
| 156 | complement (120600..  122246) | 548 | ORF158 | putative tailspike protein [Dickeya virus Limestone] YP_007237480.1 | 100-100-0 | tailspike protein |
| 157 | complement (122301..  122915) | 204 | ORF159 | putative tailspike protein [Dickeya virus Limestone] YP_007237481.1 | 100-100-1e-144 | tailspike protein, phage particle attaching domain |
| 158 | complement (122966..  124480) | 504 | ORF160 | putative tailspike protein [Dickeya virus Limestone] YP_007237482.1 | 100-100-0 | tailspike protein, particle attaching domain |
| 159 | complement (124533..  125744) | 403 | ORF161 | unknown structural protein [Dickeya virus Limestone] YP_007237483.1 | 100-100-0 | tail fiber protein |
| 160 | complement (125747.  126601) | 284 | ORF162 | putative baseplate wedge subunit gp7 [Dickeya virus Limestone] YP_007237484.1 | 100-100-0 | baseplate protein phage T4 gp7 family |
| 161 | complement (126585..  128300) | 571 | ORF163 | putative baseplate wedge subunit gp6 [Dickeya virus Limestone] YP_007237485.1 | 100-100-0 | putative baseplate protein gp6 |
| 162 | 128696..128971 | 91 | ORF164 | hypothetical protein [Dickeya virus Limestone] YP_007237486.1 | 100-100-4e-61 | hypothetical protein |
| 163 | 128961..129533 | 190 | ORF165 | hypothetical protein [Dickeya virus Limestone] YP_007237487.1 | 100-100-1e-138 | hypothetical protein |
| 164 | 129648..129806 | 52 | no | hypothetical protein [Dickeya phage JA15] ASD51351.1 | 100-100-7e-30 | hypothetical protein |
| 165 | 130734..131000 | 88 | no | No homologs |  | hypothetical protein |
| 166 | 131037..131219 | 60 | ORF167 | hypothetical protein [Dickeya virus Limestone] YP_007237489.1 | 100-100-1e-37 | hypothetical protein |
|  | 131221..131297 |  |  |  |  | tRNA-Met |
| 167 | 132332..132850 | 172 | ORF169 | unknown structural protein [Dickeya virus Limestone] YP_007237491.1 | 100-100-4e-121 | putative structural protein, possible cell puncturing protein |
| 168 | 132921..133508 | 195 | ORF170 | hypothetical protein [Dickeya virus Limestone]YP_007237492.1 | 100-100-2e-145 | hypothetical protein |
| 169 | 133578..134771 | 397 | ORF172 | putative threonine ammonia lyase [Dickeya virus Limestone] YP_007237494.1 | 99-98-0 | putative threonine ammonia lyase |
| 170 | 134811..135269 | 152 | ORF173 | unknown structural protein [Dickeya virus Limestone] YP_007237495.1 | 100-100-1e-110 | hypothetical protein |
| 171 | 135305..135763 | 152 | ORF174 | unknown structural protein [Dickeya virus Limestone]YP_007237496.1 | 100-100-1e-102 | hypothetical protein |
| 172 | complement (135789..  136163) | 124 | ORF175 | hypothetical protein [Dickeya virus Limestone] YP_007237497.1 | 100-100-1e-85 | hypothetical protein |
| 173 | complement (136165..  136725) | 186 | ORF176 | hypothetical protein [Dickeya virus Limestone] YP_007237498.1 | 100-100-1e-137 | hypothetical protein |
| 174 | complement (136767..  137339) | 190 | ORF177 | hypothetical protein [Dickeya virus Limestone] YP_007237499.1 | 100-100-6e-135 | putative holin |
| 175 | 137419..138852 | 477 | ORF178 | putative DNA polymerase gp43 [Dickeya virus Limestone] YP_007237500.1 | 100-100-0 | putative DNA polymerase |
| 176 | 138981..139859 | 292 | ORF179 | putative intron I-LimII [Dickeya virus Limestone] YP_007237501.1 | 100-100-0 | putative HNH endonuclease |
| 177 | 139973..140452 | 159 | ORF180 | DNA polymerase domain protein [Dickeya phage phiD3] AIM51287.1 | 100-100-2e-116 | putative DNA polymerase |
| 178 | complement (140439..  141281) | 280 | ORF181 | putative intron I-LimIII [Dickeya virus Limestone] YP_007237503.1 | 100-99-0 | putative HNH endonuclease |
| 179 | 141328..142461 | 377 | ORF182 | putative DNA polymerase gp43 [Dickeya virus Limestone] YP_007237504.1 | 100-100-0 | putative DNA polymerase |
| 180 | 142524..142865 | 113 | ORF183 | hypothetical protein [Dickeya virus Limestone] YP_007237505.1 | 100-100-4e-76 | hypothetical protein |
| 181 | 142862..143641 | 259 | ORF184 | putative 5'(3') deoxyribonucleotidase [Dickeya phage JA15] ASD51369.1 | 100-100-0 | putative 5'(3') deoxyribonucleotidase |
| 182 | 143651..143956 | 101 | ORF185 | putative thioredoxin [Dickeya virus Limestone]YP_007237507.1 | 100-100-3e-69 | putative thioredoxin |
| 183 | 144055..145548 | 497 | ORF186 | putative homing endonuclease F-LimXI [Dickeya virus Limestone] YP_007237508.1 | 100-100-0 | putative phosphotransferase |
| 184 | 145551..145748 | 65 | ORF187 | hypothetical protein [Dickeya virus Limestone] YP_007237509.1 | 100-98-8e-39 | hypothetical protein |
| 185 | 145745..146125 | 126 | ORF188 | hypothetical protein [Dickeya virus Limestone] YP_007237510.1 | 100-100-2e-87 | hypothetical protein |
| 186 | 146106..146204 | 32 | ORF189 | hypothetical protein [Dickeya virus Limestone] YP_007237511.1 | 100-100-7e-12 | hypothetical protein |
| 187 | 146321..147523 | 400 | ORF190 | hypothetical protein [Dickeya virus Limestone] YP_007237512.1 | 100-100-0 | hypothetical protein |
| 188 | 147560..147727 | 55 | ORF191 | hypothetical protein [Dickeya virus Limestone] YP_007237513.1 | 100-100-2e-29 | hypothetical protein |
| 189 | 147809..148021 | 70 | ORF192 | hypothetical protein [Dickeya virus Limestone] YP_007237514.1 | 100-100-5e-44 | hypothetical protein |
| 190 | 148018..148236 | 72 | ORF193 | hypothetical protein [Dickeya phage JA15] ASD51378.1 | 100-100-2e-47 | hypothetical protein |
| 191 | 148233..149348 | 371 | ORF194 | hypothetical protein [Dickeya virus Limestone] YP_007237516.1 | 100-100-0 | hypothetical protein |
| 192 | 149368..149937 | 189 | ORF195 | hypothetical protein [Dickeya virus Limestone] YP_007237517.1 | 100-100-2e-136 | hypothetical protein |
| 193 | 149942..150370 | 142 | ORF196 | hypothetical protein [Dickeya virus Limestone] YP_007237518.1 | 100-100-1e-99 | hypothetical protein |
| 194 | 150421..150798 | 125 | ORF197 | hypothetical protein HQ80_0001 [Dickeya phage phiD3] AIM51419.1 | 100-100-1e-85 | hypothetical protein |
| 195 | 150795..151241 | 148 | ORF198 | hypothetical protein [Dickeya virus Limestone] YP_007237520.1 | 100-100-2e-103 | hypothetical protein |
| 196 | 151238..151438 | 66 | ORF199 | hypothetical protein [Dickeya virus Limestone] YP_007237521.1 | 100-100-7e-40 | hypothetical protein |
| 197 | 151410..151583 | 57 | ORF200 | hypothetical protein [Dickeya virus Limestone] YP_007237522.1 | 100-100-2e-32 | hypothetical protein |
| 198 | 151594..151941 | 115 | ORF201 | hypothetical protein [Dickeya virus Limestone] YP_007237523.1 | 100-100-2e-78 | hypothetical protein |
